# 3-(4-Fluoro­phen­yl)-2-(2-naphth­yloxy)-4-oxo-5-phenyl-4,5-dihydro-3*H*-pyrrolo[3,2-*d*]pyrimidine-7-carbonitrile

**DOI:** 10.1107/S1600536809039531

**Published:** 2009-10-03

**Authors:** Jun-Kai Ma, Mei He, Yang-Gen Hu

**Affiliations:** a Institute of Medicinal Chemistry, Yunyang Medical College, Shiyan Hubei 442000, People’s Republic of China; b Department of Pharmacy, Affiliated Renmin Hospital, Yunyang Medical College, Shiyan Hubei 442000, People’s Republic of China; c Department of Pharmacy, Taihe Hospital of Yunyang Medical College and Institute of Medicinal Chemistry, Yunyang Medical College, Shiyan Hubei 442000, People’s Republic of China

## Abstract

The title compound, C_29_H_17_FN_4_O_2_, may be used as a new precursor for obtaining bioactive mol­ecules. There are two crystallographically independent mol­ecules in the asymmetric unit. The phenyl ring, 4-fluoro­phenyl ring and 2-naphth­yloxy ring are twisted with respect to the pyrrolopyrimidine ring by 52.30 (11)/49.05 (11), 80.94 (10)/88.36 (10) and 60.58 (7)/83.76 (7)°, respectively. The crystal packing is stabilized by weak C—H⋯N hydrogen bonds.

## Related literature

For the biological activity of pyrimidinone derivatives, see: Kondo *et al.* (1986 ▶) and for their pharmaceutical activity, see: Bayomi *et al.* (1986 ▶); Ding *et al.* (2004 ▶). For related structures, see: He *et al.* (2007 ▶); Hu *et al.* (2005 ▶, 2006 ▶, 2007 ▶).
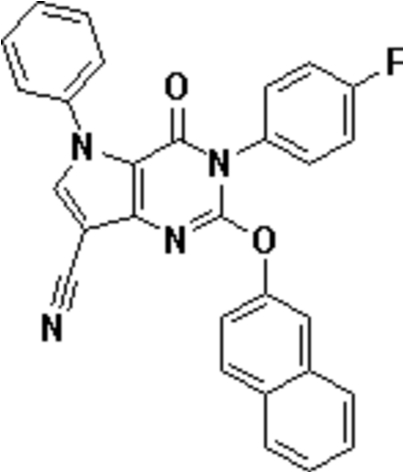


## Experimental

### 

#### Crystal data

C_29_H_17_FN_4_O_2_
*M* *_r_* = 472.47
Triclinic, 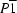

*a* = 9.8522 (16) Å
*b* = 14.549 (2) Å
*c* = 16.792 (3) Å
α = 101.755 (3)°
β = 90.840 (3)°
γ = 103.789 (3)°
*V* = 2283.5 (6) Å^3^
*Z* = 4
Mo *K*α radiation
μ = 0.09 mm^−1^
*T* = 298 K
0.16 × 0.12 × 0.10 mm

#### Data collection

Bruker SMART CCD area-detector diffractometer
Absorption correction: multi-scan (*SADABS*; Bruker, 2001 ▶) *T*_min_ = 0.985, *T*_max_ = 0.991
14836 measured reflections
8839 independent reflections
5302 reflections with *I* > 2σ(*I*)
*R*_int_ = 0.082

#### Refinement

*R*[*F* ^2^ > 2σ(*F* ^2^)] = 0.056
*wR*(*F* ^2^) = 0.129
*S* = 0.93
8839 reflections
649 parameters
H-atom parameters constrained
Δρ_max_ = 0.22 e Å^−3^
Δρ_min_ = −0.20 e Å^−3^

### 

Data collection: *SMART* (Bruker, 2001 ▶); cell refinement: *SAINT-Plus* (Bruker, 2001 ▶); data reduction: *SAINT-Plus*; program(s) used to solve structure: *SHELXS97* (Sheldrick, 2008 ▶); program(s) used to refine structure: *SHELXL97* (Sheldrick, 2008 ▶); molecular graphics: *PLATON* (Spek, 2009 ▶); software used to prepare material for publication: *SHELXL97*.

## Supplementary Material

Crystal structure: contains datablocks I, global. DOI: 10.1107/S1600536809039531/bt5077sup1.cif

Structure factors: contains datablocks I. DOI: 10.1107/S1600536809039531/bt5077Isup2.hkl

Additional supplementary materials:  crystallographic information; 3D view; checkCIF report

## Figures and Tables

**Table 1:** Hydrogen-bond geometry (Å, °)

*D*—H⋯*A*	*D*—H	H⋯*A*	*D*⋯*A*	*D*—H⋯*A*
C45—H45⋯N4^i^	0.93	2.48	3.312 (3)	150
C15—H15⋯N8^ii^	0.93	2.48	3.294 (3)	147

Symmetry codes: (i) 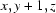; (ii) 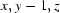.

**Fig. 1.**
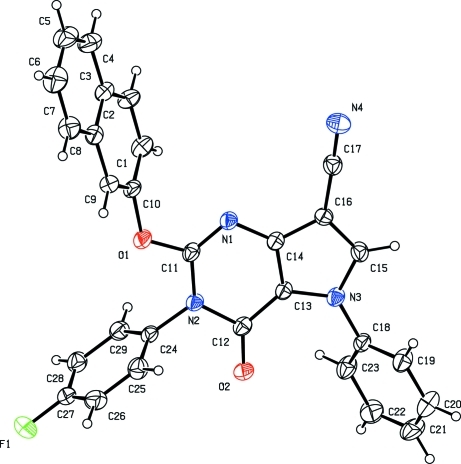
The molecular structure of the title compound, showing the atom-labeling scheme.

## supplementary crystallographic information

### Comment

Derivatives of pyrimidinone are attracting increasing attention in the synthetic chemistry community because of the important role played by such systems in many natural products, also in antibiotics and drugs (Kondo *et al.*,1986; Bayomi *et al.*, 1986; Ding *et al.*, 2004). Recently, aza-Wittig reaction of functionalized iminophosphoranes with isocyanates were applied to produce carbodiimides able to undergo a plethora of heterocyclization reactions (Hu *et al.*, 2005, 2006, 2007 and He *et al.*, 2007). As a part of our ongoing investigations on the preparation of the heterocyclic compounds, we have synthesized and structurally characterized the title compound. In the crystal structure of title compound two crystallographically independent molecules are found in the asymmetric unit. All ring atoms of pyrrolopyrimidine ring system are coplanar, with a maximum deviation of -0.024Å for atom C12. The phenyl ring and 4-fluorophenyl ring, 2-naphthalenyloxy ring are twisted with the pyrrolopyrimidine rings by 52.30 (11)/49.05 (11)°, 80.94 (10)/88.36 (10)°, 60.58 (7)/83.76 (7)°,respectively. The crystal packing is mainly stabilized by C—H···N (Table 1).

### Experimental

The title compound was obtained in excellent yield *via* aza-Wittig reaction. Crystals suitable for single-crystal X-ray diffraction were obtained by recrystallization from a mixed solvent of ethanol and dichloromethane (1:2 *v*/*v*) at room temperature.

### Refinement

All H-atoms were positioned geometrically and refined using a riding model with C—H = 0.93 Å and *U*_iso_=1.2*U*_eq_(C).

### Crystal data

**Table:**

C_29_H_17_FN_4_O_2_	*Z* = 4
*M**_r_* = 472.47	*F*(000) = 976
Triclinic, *P*1	*D*_x_ = 1.374 Mg m^−^^3^
Hall symbol: -P 1	Mo *K*α radiation, λ = 0.71073 Å
*a* = 9.8522 (16) Å	Cell parameters from 2855 reflections
*b* = 14.549 (2) Å	θ = 2.1–23.7°
*c* = 16.792 (3) Å	µ = 0.09 mm^−^^1^
α = 101.755 (3)°	*T* = 298 K
β = 90.840 (3)°	Block, colorless
γ = 103.789 (3)°	0.16 × 0.12 × 0.10 mm
*V* = 2283.5 (6) Å^3^	

### Data collection

**Table:**

Bruker SMART CCD area-detector diffractometer	8839 independent reflections
Radiation source: fine-focus sealed tube	5302 reflections with *I* > 2σ(*I*)
graphite	*R*_int_ = 0.082
φ and ω scans	θ_max_ = 26.0°, θ_min_ = 1.2°
Absorption correction: multi-scan (*SADABS*; Bruker, 2001)	*h* = −10→12
*T*_min_ = 0.985, *T*_max_ = 0.991	*k* = −17→17
14836 measured reflections	*l* = −20→20

### Refinement

**Table:**

Refinement on *F*^2^	Primary atom site location: structure-invariant direct methods
Least-squares matrix: full	Secondary atom site location: difference Fourier map
*R*[*F*^2^ > 2σ(*F*^2^)] = 0.056	Hydrogen site location: inferred from neighbouring sites
*wR*(*F*^2^) = 0.129	H-atom parameters constrained
*S* = 0.93	*w* = 1/[σ^2^(*F*_o_^2^) + (0.0375*P*)^2^] where *P* = (*F*_o_^2^ + 2*F*_c_^2^)/3
8839 reflections	(Δ/σ)_max_ = 0.001
649 parameters	Δρ_max_ = 0.22 e Å^−^^3^
0 restraints	Δρ_min_ = −0.20 e Å^−^^3^

### Special details

**Table:**

Geometry. All e.s.d.'s (except the e.s.d. in the dihedral angle between two l.s. planes) are estimated using the full covariance matrix. The cell e.s.d.'s are taken into account individually in the estimation of e.s.d.'s in distances, angles and torsion angles; correlations between e.s.d.'s in cell parameters are only used when they are defined by crystal symmetry. An approximate (isotropic) treatment of cell e.s.d.'s is used for estimating e.s.d.'s involving l.s. planes.
Refinement. Refinement of *F*^2^ against ALL reflections. The weighted *R*-factor *wR* and goodness of fit *S* are based on *F*^2^, conventional *R*-factors *R* are based on *F*, with *F* set to zero for negative *F*^2^. The threshold expression of *F*^2^ > σ(*F*^2^) is used only for calculating *R*-factors(gt) *etc*. and is not relevant to the choice of reflections for refinement. *R*-factors based on *F*^2^ are statistically about twice as large as those based on *F*, and *R*- factors based on ALL data will be even larger.

### Fractional atomic coordinates and isotropic or equivalent isotropic displacement parameters (Å^2^)

**Table:**

	*x*	*y*	*z*	*U*_iso_*/*U*_eq_	
C1	0.6132 (3)	0.62617 (17)	0.92978 (14)	0.0490 (6)	
H1	0.5394	0.5829	0.9466	0.059*	
C2	0.5894 (3)	0.67826 (17)	0.87454 (15)	0.0498 (6)	
H2	0.4987	0.6690	0.8527	0.060*	
C3	0.6992 (3)	0.74576 (16)	0.84978 (14)	0.0423 (6)	
C4	0.6778 (3)	0.80169 (19)	0.79335 (15)	0.0591 (7)	
H4	0.5879	0.7940	0.7709	0.071*	
C5	0.7866 (3)	0.8665 (2)	0.77143 (16)	0.0661 (8)	
H5	0.7702	0.9026	0.7342	0.079*	
C6	0.9217 (3)	0.8795 (2)	0.80395 (17)	0.0680 (8)	
H6	0.9950	0.9246	0.7887	0.082*	
C7	0.9477 (3)	0.82632 (18)	0.85846 (15)	0.0557 (7)	
H7	1.0387	0.8350	0.8796	0.067*	
C8	0.8371 (3)	0.75842 (16)	0.88259 (13)	0.0415 (6)	
C9	0.8599 (2)	0.70201 (16)	0.93887 (13)	0.0425 (6)	
H9	0.9499	0.7085	0.9606	0.051*	
C10	0.7502 (3)	0.63887 (16)	0.96067 (13)	0.0411 (6)	
C11	0.7475 (2)	0.49336 (16)	1.00458 (14)	0.0389 (6)	
C12	0.7632 (2)	0.35673 (16)	1.06705 (14)	0.0387 (6)	
C13	0.7014 (2)	0.30076 (15)	0.98939 (13)	0.0355 (5)	
C14	0.6725 (2)	0.34463 (15)	0.92770 (13)	0.0360 (5)	
C15	0.6130 (2)	0.18391 (17)	0.88114 (13)	0.0437 (6)	
H15	0.5804	0.1229	0.8475	0.052*	
C16	0.6166 (2)	0.27031 (16)	0.85905 (13)	0.0397 (6)	
C17	0.5731 (3)	0.28410 (17)	0.78179 (15)	0.0459 (6)	
C18	0.6789 (2)	0.12700 (16)	1.00212 (14)	0.0407 (6)	
C19	0.7474 (3)	0.05891 (18)	0.96680 (16)	0.0558 (7)	
H19	0.7841	0.0609	0.9163	0.067*	
C20	0.7614 (3)	−0.0126 (2)	1.0067 (2)	0.0724 (9)	
H20	0.8070	−0.0594	0.9830	0.087*	
C21	0.7080 (3)	−0.0144 (2)	1.0813 (2)	0.0742 (9)	
H21	0.7176	−0.0627	1.1082	0.089*	
C22	0.6406 (3)	0.0541 (2)	1.11643 (16)	0.0663 (8)	
H22	0.6061	0.0529	1.1676	0.080*	
C23	0.6239 (3)	0.12471 (17)	1.07634 (14)	0.0542 (7)	
H23	0.5758	0.1703	1.0994	0.065*	
C24	0.8394 (3)	0.52226 (15)	1.14719 (13)	0.0378 (5)	
C25	0.9802 (3)	0.54426 (17)	1.16563 (15)	0.0512 (7)	
H25	1.0385	0.5196	1.1285	0.061*	
C26	1.0361 (3)	0.60350 (19)	1.23994 (17)	0.0600 (7)	
H26	1.1319	0.6192	1.2535	0.072*	
C27	0.9470 (3)	0.63836 (17)	1.29277 (15)	0.0546 (7)	
C28	0.8073 (3)	0.61833 (17)	1.27538 (15)	0.0532 (7)	
H28	0.7498	0.6440	1.3125	0.064*	
C29	0.7516 (3)	0.55902 (16)	1.20148 (14)	0.0456 (6)	
H29	0.6557	0.5439	1.1883	0.055*	
C30	0.6220 (3)	0.69583 (19)	0.48011 (16)	0.0573 (7)	
H30	0.5594	0.7010	0.4403	0.069*	
C31	0.5805 (3)	0.63259 (19)	0.52901 (16)	0.0596 (7)	
H31	0.4890	0.5947	0.5229	0.072*	
C32	0.6745 (3)	0.62370 (17)	0.58909 (14)	0.0463 (6)	
C33	0.6349 (3)	0.55678 (19)	0.64114 (17)	0.0669 (8)	
H33	0.5437	0.5186	0.6369	0.080*	
C34	0.7312 (4)	0.5489 (2)	0.69714 (17)	0.0720 (9)	
H34	0.7052	0.5055	0.7312	0.086*	
C35	0.8680 (4)	0.6056 (2)	0.70342 (17)	0.0748 (9)	
H35	0.9328	0.5989	0.7414	0.090*	
C36	0.9086 (3)	0.6701 (2)	0.65539 (15)	0.0631 (8)	
H36	1.0002	0.7079	0.6610	0.076*	
C37	0.8132 (3)	0.68024 (16)	0.59734 (14)	0.0431 (6)	
C38	0.8538 (3)	0.74723 (17)	0.54546 (14)	0.0486 (6)	
H38	0.9445	0.7862	0.5503	0.058*	
C39	0.7584 (3)	0.75314 (17)	0.48932 (14)	0.0448 (6)	
C40	0.7721 (2)	0.90035 (16)	0.44752 (14)	0.0399 (6)	
C41	0.6952 (2)	1.02363 (15)	0.51638 (12)	0.0353 (5)	
C42	0.7355 (2)	1.07974 (15)	0.45980 (13)	0.0359 (5)	
C43	0.8047 (2)	1.04576 (16)	0.38944 (14)	0.0409 (6)	
C44	0.6360 (2)	1.07937 (16)	0.57945 (13)	0.0383 (6)	
C45	0.6438 (2)	1.16720 (17)	0.55851 (13)	0.0440 (6)	
H45	0.6127	1.2181	0.5892	0.053*	
C46	0.5761 (2)	1.05029 (17)	0.64986 (14)	0.0430 (6)	
C47	0.7274 (2)	1.25012 (17)	0.44751 (14)	0.0425 (6)	
C48	0.7886 (3)	1.34087 (17)	0.49336 (16)	0.0539 (7)	
H48	0.8155	1.3489	0.5482	0.065*	
C49	0.8099 (3)	1.41970 (19)	0.4573 (2)	0.0702 (9)	
H49	0.8496	1.4815	0.4880	0.084*	
C50	0.7721 (3)	1.4067 (2)	0.3757 (2)	0.0780 (10)	
H50	0.7883	1.4597	0.3511	0.094*	
C51	0.7108 (3)	1.3161 (2)	0.33079 (18)	0.0715 (9)	
H51	0.6853	1.3080	0.2758	0.086*	
C52	0.6866 (3)	1.23672 (19)	0.36637 (15)	0.0554 (7)	
H52	0.6436	1.1753	0.3361	0.067*	
C53	0.8782 (2)	0.90428 (15)	0.31605 (13)	0.0363 (5)	
C54	0.7934 (3)	0.85468 (17)	0.24827 (14)	0.0475 (6)	
H54	0.6977	0.8507	0.2478	0.057*	
C55	0.8494 (3)	0.81039 (17)	0.18027 (14)	0.0494 (6)	
H55	0.7927	0.7759	0.1339	0.059*	
C56	0.9890 (3)	0.81875 (17)	0.18328 (14)	0.0440 (6)	
C57	1.0759 (3)	0.86866 (19)	0.24921 (15)	0.0582 (7)	
H57	1.1718	0.8736	0.2487	0.070*	
C58	1.0190 (3)	0.91202 (19)	0.31723 (14)	0.0528 (7)	
H58	1.0762	0.9462	0.3635	0.063*	
F1	1.00295 (19)	0.69609 (12)	1.36557 (9)	0.0862 (5)	
F2	1.04529 (16)	0.77395 (11)	0.11711 (8)	0.0686 (4)	
N1	0.6958 (2)	0.44287 (13)	0.93398 (11)	0.0402 (5)	
N2	0.78044 (19)	0.45772 (12)	1.07043 (10)	0.0375 (5)	
N3	0.66453 (19)	0.20168 (13)	0.95990 (11)	0.0403 (5)	
N4	0.5398 (3)	0.29764 (17)	0.72066 (13)	0.0658 (7)	
N5	0.71351 (19)	0.93223 (13)	0.51206 (11)	0.0401 (5)	
N6	0.81678 (19)	0.94956 (13)	0.38718 (10)	0.0386 (5)	
N7	0.7039 (2)	1.16818 (13)	0.48610 (11)	0.0418 (5)	
N8	0.5282 (2)	1.02686 (16)	0.70593 (12)	0.0638 (7)	
O1	0.77772 (18)	0.59064 (11)	1.02224 (9)	0.0511 (4)	
O2	0.79870 (18)	0.32927 (11)	1.12612 (9)	0.0526 (5)	
O3	0.80000 (19)	0.81309 (12)	0.43311 (9)	0.0562 (5)	
O4	0.85016 (19)	1.08711 (12)	0.33598 (10)	0.0592 (5)	

### Atomic displacement parameters (Å^2^)

**Table:**

	*U*^11^	*U*^22^	*U*^33^	*U*^12^	*U*^13^	*U*^23^
C1	0.0468 (16)	0.0467 (15)	0.0597 (17)	0.0125 (12)	0.0161 (13)	0.0236 (13)
C2	0.0431 (15)	0.0522 (16)	0.0632 (17)	0.0209 (12)	0.0056 (13)	0.0218 (14)
C3	0.0512 (16)	0.0401 (14)	0.0428 (14)	0.0196 (12)	0.0125 (12)	0.0148 (11)
C4	0.0720 (19)	0.0639 (18)	0.0542 (17)	0.0303 (15)	0.0083 (15)	0.0253 (14)
C5	0.089 (2)	0.0643 (19)	0.0574 (18)	0.0242 (17)	0.0155 (17)	0.0354 (15)
C6	0.082 (2)	0.0605 (18)	0.066 (2)	0.0102 (16)	0.0277 (17)	0.0307 (16)
C7	0.0564 (17)	0.0529 (16)	0.0573 (17)	0.0063 (13)	0.0110 (13)	0.0187 (14)
C8	0.0521 (16)	0.0382 (13)	0.0359 (13)	0.0129 (11)	0.0108 (12)	0.0092 (11)
C9	0.0462 (15)	0.0412 (14)	0.0426 (14)	0.0142 (12)	−0.0002 (11)	0.0108 (11)
C10	0.0607 (17)	0.0332 (13)	0.0354 (13)	0.0178 (12)	0.0058 (12)	0.0130 (11)
C11	0.0498 (15)	0.0317 (13)	0.0389 (14)	0.0122 (11)	0.0104 (12)	0.0129 (11)
C12	0.0433 (14)	0.0375 (13)	0.0398 (14)	0.0118 (11)	0.0120 (11)	0.0156 (11)
C13	0.0444 (14)	0.0321 (12)	0.0335 (13)	0.0139 (10)	0.0095 (11)	0.0095 (10)
C14	0.0421 (14)	0.0341 (13)	0.0347 (13)	0.0123 (10)	0.0093 (11)	0.0098 (11)
C15	0.0567 (16)	0.0386 (14)	0.0337 (14)	0.0103 (12)	0.0058 (12)	0.0045 (11)
C16	0.0490 (15)	0.0408 (14)	0.0312 (13)	0.0140 (11)	0.0033 (11)	0.0088 (11)
C17	0.0528 (16)	0.0426 (15)	0.0413 (15)	0.0108 (12)	0.0064 (13)	0.0075 (12)
C18	0.0509 (15)	0.0311 (13)	0.0422 (14)	0.0113 (11)	0.0041 (12)	0.0112 (11)
C19	0.0741 (19)	0.0441 (15)	0.0544 (16)	0.0224 (14)	0.0172 (14)	0.0127 (13)
C20	0.087 (2)	0.0472 (17)	0.093 (2)	0.0297 (16)	0.0126 (19)	0.0214 (17)
C21	0.093 (2)	0.0516 (18)	0.086 (2)	0.0143 (17)	−0.0049 (19)	0.0391 (17)
C22	0.093 (2)	0.0533 (18)	0.0549 (18)	0.0081 (16)	0.0102 (16)	0.0266 (15)
C23	0.0732 (19)	0.0423 (15)	0.0527 (16)	0.0175 (13)	0.0202 (14)	0.0181 (13)
C24	0.0497 (15)	0.0302 (12)	0.0365 (13)	0.0113 (11)	0.0033 (12)	0.0126 (11)
C25	0.0485 (17)	0.0499 (15)	0.0562 (17)	0.0164 (13)	0.0072 (13)	0.0081 (13)
C26	0.0545 (17)	0.0563 (17)	0.069 (2)	0.0145 (14)	−0.0126 (15)	0.0141 (15)
C27	0.084 (2)	0.0390 (15)	0.0442 (16)	0.0216 (15)	−0.0121 (16)	0.0091 (13)
C28	0.074 (2)	0.0480 (16)	0.0422 (16)	0.0254 (14)	0.0077 (14)	0.0091 (13)
C29	0.0507 (15)	0.0458 (15)	0.0447 (15)	0.0185 (12)	0.0055 (13)	0.0116 (12)
C30	0.0614 (19)	0.0623 (18)	0.0586 (17)	0.0276 (15)	0.0073 (14)	0.0220 (15)
C31	0.0504 (17)	0.0607 (18)	0.0686 (19)	0.0126 (14)	0.0064 (15)	0.0169 (15)
C32	0.0577 (17)	0.0413 (14)	0.0444 (15)	0.0194 (13)	0.0098 (13)	0.0104 (12)
C33	0.086 (2)	0.0537 (17)	0.0650 (19)	0.0168 (16)	0.0207 (17)	0.0217 (15)
C34	0.114 (3)	0.0609 (19)	0.060 (2)	0.036 (2)	0.030 (2)	0.0369 (16)
C35	0.105 (3)	0.076 (2)	0.062 (2)	0.043 (2)	0.0090 (18)	0.0303 (17)
C36	0.072 (2)	0.0658 (19)	0.0578 (18)	0.0257 (16)	0.0009 (15)	0.0180 (15)
C37	0.0544 (16)	0.0411 (14)	0.0395 (14)	0.0206 (12)	0.0021 (12)	0.0111 (11)
C38	0.0579 (17)	0.0432 (14)	0.0488 (16)	0.0176 (12)	0.0097 (13)	0.0125 (12)
C39	0.0640 (18)	0.0391 (14)	0.0416 (14)	0.0259 (13)	0.0164 (13)	0.0151 (12)
C40	0.0511 (15)	0.0372 (14)	0.0368 (13)	0.0152 (11)	0.0069 (12)	0.0149 (11)
C41	0.0410 (13)	0.0364 (13)	0.0303 (12)	0.0118 (11)	0.0002 (10)	0.0086 (10)
C42	0.0454 (14)	0.0341 (13)	0.0330 (12)	0.0153 (11)	0.0085 (11)	0.0115 (10)
C43	0.0485 (15)	0.0407 (14)	0.0386 (14)	0.0143 (11)	0.0097 (12)	0.0158 (11)
C44	0.0443 (14)	0.0432 (14)	0.0312 (13)	0.0147 (11)	0.0052 (11)	0.0117 (11)
C45	0.0515 (15)	0.0475 (15)	0.0375 (14)	0.0212 (12)	0.0078 (12)	0.0081 (11)
C46	0.0481 (15)	0.0458 (14)	0.0355 (14)	0.0143 (12)	0.0039 (12)	0.0060 (12)
C47	0.0462 (15)	0.0423 (14)	0.0463 (15)	0.0168 (11)	0.0061 (12)	0.0188 (12)
C48	0.0582 (17)	0.0429 (16)	0.0646 (17)	0.0188 (13)	0.0005 (14)	0.0131 (14)
C49	0.0590 (19)	0.0391 (16)	0.115 (3)	0.0128 (13)	0.0037 (18)	0.0214 (17)
C50	0.069 (2)	0.071 (2)	0.117 (3)	0.0235 (18)	0.018 (2)	0.063 (2)
C51	0.074 (2)	0.087 (2)	0.072 (2)	0.0259 (18)	0.0074 (17)	0.0509 (19)
C52	0.0633 (18)	0.0545 (16)	0.0531 (17)	0.0146 (14)	0.0022 (14)	0.0219 (14)
C53	0.0438 (15)	0.0353 (13)	0.0332 (13)	0.0119 (11)	0.0076 (11)	0.0121 (10)
C54	0.0414 (14)	0.0525 (15)	0.0488 (15)	0.0128 (12)	0.0048 (13)	0.0093 (13)
C55	0.0559 (17)	0.0500 (15)	0.0382 (14)	0.0105 (13)	−0.0016 (13)	0.0033 (12)
C56	0.0556 (17)	0.0419 (14)	0.0384 (14)	0.0172 (12)	0.0152 (13)	0.0102 (12)
C57	0.0430 (16)	0.079 (2)	0.0510 (17)	0.0196 (14)	0.0053 (14)	0.0048 (15)
C58	0.0479 (17)	0.0681 (18)	0.0380 (15)	0.0143 (13)	−0.0021 (12)	0.0016 (13)
F1	0.1202 (15)	0.0762 (11)	0.0562 (10)	0.0345 (10)	−0.0334 (10)	−0.0087 (9)
F2	0.0802 (11)	0.0779 (11)	0.0503 (9)	0.0310 (9)	0.0238 (8)	0.0049 (8)
N1	0.0551 (13)	0.0349 (11)	0.0331 (11)	0.0134 (9)	0.0049 (9)	0.0099 (9)
N2	0.0507 (12)	0.0346 (11)	0.0310 (11)	0.0141 (9)	0.0063 (9)	0.0112 (9)
N3	0.0518 (12)	0.0355 (11)	0.0366 (11)	0.0123 (9)	0.0088 (9)	0.0120 (9)
N4	0.0884 (18)	0.0704 (16)	0.0422 (13)	0.0212 (13)	−0.0010 (13)	0.0186 (12)
N5	0.0531 (13)	0.0403 (11)	0.0334 (11)	0.0188 (9)	0.0093 (10)	0.0141 (9)
N6	0.0506 (12)	0.0379 (11)	0.0330 (11)	0.0172 (9)	0.0100 (9)	0.0130 (9)
N7	0.0557 (13)	0.0379 (11)	0.0382 (11)	0.0184 (10)	0.0088 (10)	0.0137 (9)
N8	0.0809 (17)	0.0674 (15)	0.0411 (13)	0.0101 (13)	0.0171 (12)	0.0155 (12)
O1	0.0797 (13)	0.0349 (9)	0.0416 (10)	0.0162 (9)	−0.0006 (9)	0.0129 (8)
O2	0.0777 (13)	0.0433 (10)	0.0414 (10)	0.0175 (9)	−0.0042 (9)	0.0167 (8)
O3	0.0910 (14)	0.0457 (10)	0.0494 (10)	0.0374 (10)	0.0275 (10)	0.0240 (8)
O4	0.0870 (14)	0.0508 (11)	0.0529 (11)	0.0271 (10)	0.0350 (10)	0.0267 (9)

### Geometric parameters (Å, °)

**Table:**

C1—C2	1.362 (3)	C30—C31	1.352 (3)
C1—C10	1.396 (3)	C30—C39	1.390 (3)
C1—H1	0.9300	C30—H30	0.9300
C2—C3	1.407 (3)	C31—C32	1.406 (3)
C2—H2	0.9300	C31—H31	0.9300
C3—C4	1.412 (3)	C32—C37	1.405 (3)
C3—C8	1.414 (3)	C32—C33	1.428 (3)
C4—C5	1.358 (4)	C33—C34	1.366 (4)
C4—H4	0.9300	C33—H33	0.9300
C5—C6	1.387 (4)	C34—C35	1.392 (4)
C5—H5	0.9300	C34—H34	0.9300
C6—C7	1.371 (3)	C35—C36	1.354 (4)
C6—H6	0.9300	C35—H35	0.9300
C7—C8	1.411 (3)	C36—C37	1.397 (3)
C7—H7	0.9300	C36—H36	0.9300
C8—C9	1.420 (3)	C37—C38	1.428 (3)
C9—C10	1.349 (3)	C38—C39	1.351 (3)
C9—H9	0.9300	C38—H38	0.9300
C10—O1	1.419 (2)	C39—O3	1.413 (3)
C11—N1	1.286 (3)	C40—N5	1.290 (3)
C11—O1	1.344 (2)	C40—O3	1.338 (3)
C11—N2	1.378 (3)	C40—N6	1.376 (3)
C12—O2	1.218 (2)	C41—N5	1.372 (3)
C12—N2	1.427 (3)	C41—C42	1.378 (3)
C12—C13	1.429 (3)	C41—C44	1.419 (3)
C13—C14	1.382 (3)	C42—N7	1.383 (3)
C13—N3	1.382 (3)	C42—C43	1.432 (3)
C14—N1	1.374 (3)	C43—O4	1.213 (2)
C14—C16	1.411 (3)	C43—N6	1.425 (3)
C15—N3	1.361 (3)	C44—C45	1.378 (3)
C15—C16	1.374 (3)	C44—C46	1.425 (3)
C15—H15	0.9300	C45—N7	1.361 (3)
C16—C17	1.427 (3)	C45—H45	0.9300
C17—N4	1.142 (3)	C46—N8	1.137 (3)
C18—C23	1.370 (3)	C47—C48	1.376 (3)
C18—C19	1.371 (3)	C47—C52	1.376 (3)
C18—N3	1.445 (3)	C47—N7	1.443 (3)
C19—C20	1.378 (3)	C48—C49	1.378 (3)
C19—H19	0.9300	C48—H48	0.9300
C20—C21	1.368 (4)	C49—C50	1.377 (4)
C20—H20	0.9300	C49—H49	0.9300
C21—C22	1.367 (4)	C50—C51	1.368 (4)
C21—H21	0.9300	C50—H50	0.9300
C22—C23	1.377 (3)	C51—C52	1.379 (3)
C22—H22	0.9300	C51—H51	0.9300
C23—H23	0.9300	C52—H52	0.9300
C24—C25	1.363 (3)	C53—C58	1.363 (3)
C24—C29	1.382 (3)	C53—C54	1.366 (3)
C24—N2	1.451 (3)	C53—N6	1.455 (3)
C25—C26	1.385 (3)	C54—C55	1.383 (3)
C25—H25	0.9300	C54—H54	0.9300
C26—C27	1.366 (4)	C55—C56	1.351 (3)
C26—H26	0.9300	C55—H55	0.9300
C27—C28	1.352 (4)	C56—C57	1.355 (3)
C27—F1	1.358 (3)	C56—F2	1.365 (2)
C28—C29	1.380 (3)	C57—C58	1.383 (3)
C28—H28	0.9300	C57—H57	0.9300
C29—H29	0.9300	C58—H58	0.9300
			
C2—C1—C10	118.8 (2)	C31—C32—C33	122.1 (3)
C2—C1—H1	120.6	C34—C33—C32	119.9 (3)
C10—C1—H1	120.6	C34—C33—H33	120.0
C1—C2—C3	121.4 (2)	C32—C33—H33	120.0
C1—C2—H2	119.3	C33—C34—C35	120.2 (3)
C3—C2—H2	119.3	C33—C34—H34	119.9
C2—C3—C4	122.8 (2)	C35—C34—H34	119.9
C2—C3—C8	118.9 (2)	C36—C35—C34	121.3 (3)
C4—C3—C8	118.4 (2)	C36—C35—H35	119.4
C5—C4—C3	120.9 (3)	C34—C35—H35	119.4
C5—C4—H4	119.5	C35—C36—C37	120.1 (3)
C3—C4—H4	119.5	C35—C36—H36	119.9
C4—C5—C6	120.8 (3)	C37—C36—H36	119.9
C4—C5—H5	119.6	C36—C37—C32	120.0 (2)
C6—C5—H5	119.6	C36—C37—C38	121.2 (2)
C7—C6—C5	120.3 (3)	C32—C37—C38	118.8 (2)
C7—C6—H6	119.8	C39—C38—C37	119.0 (2)
C5—C6—H6	119.8	C39—C38—H38	120.5
C6—C7—C8	120.3 (3)	C37—C38—H38	120.5
C6—C7—H7	119.8	C38—C39—C30	122.2 (2)
C8—C7—H7	119.8	C38—C39—O3	119.6 (2)
C7—C8—C3	119.2 (2)	C30—C39—O3	118.0 (2)
C7—C8—C9	122.0 (2)	N5—C40—O3	122.2 (2)
C3—C8—C9	118.8 (2)	N5—C40—N6	126.2 (2)
C10—C9—C8	119.6 (2)	O3—C40—N6	111.60 (19)
C10—C9—H9	120.2	N5—C41—C42	125.5 (2)
C8—C9—H9	120.2	N5—C41—C44	127.5 (2)
C9—C10—C1	122.5 (2)	C42—C41—C44	107.03 (19)
C9—C10—O1	117.0 (2)	C41—C42—N7	108.81 (18)
C1—C10—O1	120.3 (2)	C41—C42—C43	121.1 (2)
N1—C11—O1	122.4 (2)	N7—C42—C43	129.97 (19)
N1—C11—N2	126.2 (2)	O4—C43—N6	120.3 (2)
O1—C11—N2	111.41 (19)	O4—C43—C42	129.0 (2)
O2—C12—N2	120.1 (2)	N6—C43—C42	110.68 (18)
O2—C12—C13	129.1 (2)	C45—C44—C41	106.64 (19)
N2—C12—C13	110.78 (19)	C45—C44—C46	126.1 (2)
C14—C13—N3	108.11 (19)	C41—C44—C46	127.2 (2)
C14—C13—C12	121.3 (2)	N7—C45—C44	109.7 (2)
N3—C13—C12	130.6 (2)	N7—C45—H45	125.1
N1—C14—C13	125.1 (2)	C44—C45—H45	125.1
N1—C14—C16	127.6 (2)	N8—C46—C44	179.8 (3)
C13—C14—C16	107.3 (2)	C48—C47—C52	121.2 (2)
N3—C15—C16	109.3 (2)	C48—C47—N7	118.9 (2)
N3—C15—H15	125.3	C52—C47—N7	119.8 (2)
C16—C15—H15	125.3	C47—C48—C49	119.4 (3)
C15—C16—C14	106.97 (19)	C47—C48—H48	120.3
C15—C16—C17	127.4 (2)	C49—C48—H48	120.3
C14—C16—C17	125.7 (2)	C50—C49—C48	119.8 (3)
N4—C17—C16	178.2 (3)	C50—C49—H49	120.1
C23—C18—C19	120.9 (2)	C48—C49—H49	120.1
C23—C18—N3	119.9 (2)	C51—C50—C49	120.3 (3)
C19—C18—N3	119.2 (2)	C51—C50—H50	119.8
C18—C19—C20	119.5 (2)	C49—C50—H50	119.8
C18—C19—H19	120.2	C50—C51—C52	120.5 (3)
C20—C19—H19	120.2	C50—C51—H51	119.7
C21—C20—C19	119.8 (3)	C52—C51—H51	119.7
C21—C20—H20	120.1	C47—C52—C51	118.8 (3)
C19—C20—H20	120.1	C47—C52—H52	120.6
C22—C21—C20	120.4 (3)	C51—C52—H52	120.6
C22—C21—H21	119.8	C58—C53—C54	120.7 (2)
C20—C21—H21	119.8	C58—C53—N6	120.2 (2)
C21—C22—C23	120.2 (3)	C54—C53—N6	119.2 (2)
C21—C22—H22	119.9	C53—C54—C55	120.1 (2)
C23—C22—H22	119.9	C53—C54—H54	119.9
C18—C23—C22	119.2 (2)	C55—C54—H54	119.9
C18—C23—H23	120.4	C56—C55—C54	118.0 (2)
C22—C23—H23	120.4	C56—C55—H55	121.0
C25—C24—C29	120.8 (2)	C54—C55—H55	121.0
C25—C24—N2	119.7 (2)	C55—C56—C57	123.2 (2)
C29—C24—N2	119.5 (2)	C55—C56—F2	118.6 (2)
C24—C25—C26	119.7 (2)	C57—C56—F2	118.2 (2)
C24—C25—H25	120.2	C56—C57—C58	118.5 (2)
C26—C25—H25	120.2	C56—C57—H57	120.8
C27—C26—C25	118.4 (3)	C58—C57—H57	120.8
C27—C26—H26	120.8	C53—C58—C57	119.6 (2)
C25—C26—H26	120.8	C53—C58—H58	120.2
C28—C27—F1	119.3 (3)	C57—C58—H58	120.2
C28—C27—C26	122.9 (2)	C11—N1—C14	113.77 (18)
F1—C27—C26	117.8 (3)	C11—N2—C12	122.77 (19)
C27—C28—C29	118.7 (2)	C11—N2—C24	121.16 (18)
C27—C28—H28	120.7	C12—N2—C24	116.04 (17)
C29—C28—H28	120.7	C15—N3—C13	108.28 (18)
C28—C29—C24	119.5 (2)	C15—N3—C18	123.98 (18)
C28—C29—H29	120.2	C13—N3—C18	127.73 (19)
C24—C29—H29	120.2	C40—N5—C41	113.49 (19)
C31—C30—C39	120.0 (3)	C40—N6—C43	122.93 (18)
C31—C30—H30	120.0	C40—N6—C53	120.83 (18)
C39—C30—H30	120.0	C43—N6—C53	116.23 (17)
C30—C31—C32	120.5 (3)	C45—N7—C42	107.81 (18)
C30—C31—H31	119.7	C45—N7—C47	123.35 (19)
C32—C31—H31	119.7	C42—N7—C47	128.84 (18)
C37—C32—C31	119.5 (2)	C11—O1—C10	118.48 (17)
C37—C32—C33	118.4 (2)	C40—O3—C39	118.28 (17)
			
C10—C1—C2—C3	−1.5 (4)	N5—C41—C44—C46	−3.6 (4)
C1—C2—C3—C4	−179.2 (2)	C42—C41—C44—C46	178.0 (2)
C1—C2—C3—C8	0.8 (3)	C41—C44—C45—N7	0.2 (3)
C2—C3—C4—C5	179.6 (2)	C46—C44—C45—N7	−178.1 (2)
C8—C3—C4—C5	−0.4 (4)	C45—C44—C46—N8	160 (100)
C3—C4—C5—C6	0.0 (4)	C41—C44—C46—N8	−18 (100)
C4—C5—C6—C7	0.5 (4)	C52—C47—C48—C49	−0.1 (4)
C5—C6—C7—C8	−0.6 (4)	N7—C47—C48—C49	−179.2 (2)
C6—C7—C8—C3	0.1 (4)	C47—C48—C49—C50	−1.2 (4)
C6—C7—C8—C9	180.0 (2)	C48—C49—C50—C51	1.4 (5)
C2—C3—C8—C7	−179.6 (2)	C49—C50—C51—C52	−0.3 (5)
C4—C3—C8—C7	0.3 (3)	C48—C47—C52—C51	1.3 (4)
C2—C3—C8—C9	0.5 (3)	N7—C47—C52—C51	−179.7 (2)
C4—C3—C8—C9	−179.5 (2)	C50—C51—C52—C47	−1.1 (4)
C7—C8—C9—C10	179.2 (2)	C58—C53—C54—C55	1.0 (3)
C3—C8—C9—C10	−0.9 (3)	N6—C53—C54—C55	−179.4 (2)
C8—C9—C10—C1	0.1 (3)	C53—C54—C55—C56	−0.6 (3)
C8—C9—C10—O1	−174.77 (18)	C54—C55—C56—C57	−0.4 (4)
C2—C1—C10—C9	1.1 (4)	C54—C55—C56—F2	178.9 (2)
C2—C1—C10—O1	175.8 (2)	C55—C56—C57—C58	1.0 (4)
O2—C12—C13—C14	−177.5 (2)	F2—C56—C57—C58	−178.3 (2)
N2—C12—C13—C14	3.2 (3)	C54—C53—C58—C57	−0.4 (4)
O2—C12—C13—N3	0.3 (4)	N6—C53—C58—C57	−180.0 (2)
N2—C12—C13—N3	−179.0 (2)	C56—C57—C58—C53	−0.6 (4)
N3—C13—C14—N1	−179.70 (19)	O1—C11—N1—C14	179.8 (2)
C12—C13—C14—N1	−1.5 (3)	N2—C11—N1—C14	0.6 (3)
N3—C13—C14—C16	0.0 (2)	C13—C14—N1—C11	−0.6 (3)
C12—C13—C14—C16	178.3 (2)	C16—C14—N1—C11	179.7 (2)
N3—C15—C16—C14	−0.2 (3)	N1—C11—N2—C12	1.6 (4)
N3—C15—C16—C17	179.6 (2)	O1—C11—N2—C12	−177.76 (18)
N1—C14—C16—C15	179.8 (2)	N1—C11—N2—C24	179.6 (2)
C13—C14—C16—C15	0.1 (3)	O1—C11—N2—C24	0.3 (3)
N1—C14—C16—C17	0.0 (4)	O2—C12—N2—C11	177.3 (2)
C13—C14—C16—C17	−179.7 (2)	C13—C12—N2—C11	−3.3 (3)
C15—C16—C17—N4	−172 (9)	O2—C12—N2—C24	−0.8 (3)
C14—C16—C17—N4	8(9)	C13—C12—N2—C24	178.60 (18)
C23—C18—C19—C20	−0.2 (4)	C25—C24—N2—C11	−99.4 (3)
N3—C18—C19—C20	−180.0 (2)	C29—C24—N2—C11	82.0 (3)
C18—C19—C20—C21	−0.5 (4)	C25—C24—N2—C12	78.7 (3)
C19—C20—C21—C22	0.1 (5)	C29—C24—N2—C12	−99.9 (2)
C20—C21—C22—C23	1.1 (5)	C16—C15—N3—C13	0.3 (3)
C19—C18—C23—C22	1.4 (4)	C16—C15—N3—C18	−178.5 (2)
N3—C18—C23—C22	−178.9 (2)	C14—C13—N3—C15	−0.2 (2)
C21—C22—C23—C18	−1.9 (4)	C12—C13—N3—C15	−178.2 (2)
C29—C24—C25—C26	0.6 (4)	C14—C13—N3—C18	178.5 (2)
N2—C24—C25—C26	−178.0 (2)	C12—C13—N3—C18	0.5 (4)
C24—C25—C26—C27	−0.1 (4)	C23—C18—N3—C15	−128.1 (2)
C25—C26—C27—C28	−0.6 (4)	C19—C18—N3—C15	51.6 (3)
C25—C26—C27—F1	179.8 (2)	C23—C18—N3—C13	53.4 (3)
F1—C27—C28—C29	−179.5 (2)	C19—C18—N3—C13	−126.9 (2)
C26—C27—C28—C29	0.9 (4)	O3—C40—N5—C41	179.2 (2)
C27—C28—C29—C24	−0.4 (3)	N6—C40—N5—C41	1.1 (3)
C25—C24—C29—C28	−0.3 (3)	C42—C41—N5—C40	−1.0 (3)
N2—C24—C29—C28	178.30 (19)	C44—C41—N5—C40	−179.2 (2)
C39—C30—C31—C32	−0.4 (4)	N5—C40—N6—C43	0.8 (4)
C30—C31—C32—C37	−0.9 (4)	O3—C40—N6—C43	−177.44 (19)
C30—C31—C32—C33	−179.1 (2)	N5—C40—N6—C53	−178.4 (2)
C37—C32—C33—C34	−0.1 (4)	O3—C40—N6—C53	3.4 (3)
C31—C32—C33—C34	178.2 (2)	O4—C43—N6—C40	177.5 (2)
C32—C33—C34—C35	−0.3 (4)	C42—C43—N6—C40	−2.6 (3)
C33—C34—C35—C36	0.8 (4)	O4—C43—N6—C53	−3.3 (3)
C34—C35—C36—C37	−0.8 (4)	C42—C43—N6—C53	176.62 (19)
C35—C36—C37—C32	0.4 (4)	C58—C53—N6—C40	−89.2 (3)
C35—C36—C37—C38	−179.4 (2)	C54—C53—N6—C40	91.2 (3)
C31—C32—C37—C36	−178.3 (2)	C58—C53—N6—C43	91.6 (3)
C33—C32—C37—C36	0.1 (3)	C54—C53—N6—C43	−88.0 (2)
C31—C32—C37—C38	1.5 (3)	C44—C45—N7—C42	0.0 (3)
C33—C32—C37—C38	179.9 (2)	C44—C45—N7—C47	−179.8 (2)
C36—C37—C38—C39	178.8 (2)	C41—C42—N7—C45	−0.2 (2)
C32—C37—C38—C39	−1.0 (3)	C43—C42—N7—C45	−177.0 (2)
C37—C38—C39—C30	−0.3 (4)	C41—C42—N7—C47	179.6 (2)
C37—C38—C39—O3	−174.76 (18)	C43—C42—N7—C47	2.8 (4)
C31—C30—C39—C38	1.0 (4)	C48—C47—N7—C45	47.9 (3)
C31—C30—C39—O3	175.6 (2)	C52—C47—N7—C45	−131.1 (2)
N5—C41—C42—N7	−178.1 (2)	C48—C47—N7—C42	−131.9 (2)
C44—C41—C42—N7	0.3 (2)	C52—C47—N7—C42	49.1 (3)
N5—C41—C42—C43	−1.0 (3)	N1—C11—O1—C10	0.5 (3)
C44—C41—C42—C43	177.5 (2)	N2—C11—O1—C10	179.88 (19)
C41—C42—C43—O4	−177.5 (2)	C9—C10—O1—C11	−121.3 (2)
N7—C42—C43—O4	−1.0 (4)	C1—C10—O1—C11	63.7 (3)
C41—C42—C43—N6	2.6 (3)	N5—C40—O3—C39	2.5 (3)
N7—C42—C43—N6	179.1 (2)	N6—C40—O3—C39	−179.12 (19)
N5—C41—C44—C45	178.1 (2)	C38—C39—O3—C40	−100.2 (3)
C42—C41—C44—C45	−0.3 (2)	C30—C39—O3—C40	85.1 (3)

### Hydrogen-bond geometry (Å, °)

**Table:**

*D*—H···*A*	*D*—H	H···*A*	*D*···*A*	*D*—H···*A*
C45—H45···N4^i^	0.93	2.48	3.312 (3)	150
C15—H15···N8^ii^	0.93	2.48	3.294 (3)	147

Symmetry codes: (i) *x*, *y*+1, *z*; (ii) *x*, *y*−1, *z*.
